# HDAC6 inhibitor ACY1215 inhibits the activation of NLRP3 inflammasome in acute liver failure by regulating the ATM/F‐actin signalling pathway

**DOI:** 10.1111/jcmm.16751

**Published:** 2021-06-27

**Authors:** Qian Chen, Yao Wang, Fangzhou Jiao, Pan Cao, Chunxia Shi, Maohua Pei, Luwen Wang, Zuojiong Gong

**Affiliations:** ^1^ Department of Infectious Diseases Renmin Hospital of Wuhan University China

**Keywords:** acute liver failure, ACY1215, ATM, F‐actin, NLRP3

## Abstract

Acute liver failure (ALF) is a rare and critical medical condition. This study was designed to investigate the protective effects and underlying mechanism of ACY1215 in ALF mice. Our findings suggested that ACY1215 treatment ameliorates the pathological hepatic damage of ALF and decreases the serum levels of ALT and AST. Furthermore, ACY1215 pretreatment increased the level of ATM, γ‐H2AX, Chk2, p53, p21, F‐actin and vinculin in ALF. Moreover, ACY1215 inhibited the level of NLRP3, ASC, caspase‐1, IL‐1β and IL‐18 in ALF. The ATM inhibitor KU55933 could decrease the level of ATM, γ‐H2AX, Chk2, p53, p21, F‐actin and vinculin in ALF with ACY1215 pretreatment. The F‐actin inhibitor cytochalasin B decreased the level of F‐actin and vinculin in ALF with ACY1215 pretreatment. However, cytochalasin B had no effect on protein levels of ATM, Chk2, p53 and p21 in ALF with ACY1215 pretreatment. Cytochalasin B could dramatically increase the level of NLRP3, ASC, caspase‐1, IL‐1β and IL‐18 in ALF with ACY1215 pretreatment. These results indicated that ACY1215 exhibited hepatoprotective properties, which was associated with the inhibition of NLRP3 inflammasome, and this effect of ACY1215 was connected with upregulation of the ATM/F‐actin mediated signalling pathways.

## 1 INTRODUCTION

Acute liver failure (ALF) is a complex and life‐threatening disease with disparate aetiology.^1^ It can progress to multiple organ dysfunction syndrome in a short time. There is currently no specific treatment for ALF. Of note, the pathogenesis of ALF encompasses not only direct liver damage but immunologically mediated processes that are triggered by multifarious causes.^2^ Accumulating evidence indicates that the activation of inflammasome plays a pivotal role in ALF.^3^ For example, the previous study has uncovered that IL‐1β overproduction due to inflammasome activation is considered as the dominating contributor for ALF.^4^ The nucleotide‐binding domain and leucine‐rich repeat‐containing family pyrin domain containing 3 (NLRP3) inflammasome has been shown to be existed in hepatocytes, Kupffer cells, hepatic stellate cells and sinusoidal endothelial cells.^5^ Hepatitis C virus (HCV) can trigger NLRP3 inflammasome activation from hepatic macrophages, to drive the hepatic inflammatory response.^6^ Moreover, NLRP3 inflammasome blockade alleviates liver inflammation and fibrosis in experimental mice with non‐alcoholic fatty liver disease (NAFLD).^7^

Previous studies have found that hepatocellular double‐stranded DNA structure is broken in ALF.^8^ Ataxia telangiectasia mutated (ATM), a core component of the DNA repair system, plays critical roles in the pathological features of liver failure as well.^9^ ATM signalling pathway is activated to enhance the homologous recombination repair pathway upon DNA double‐strand breaks. Besides, the ATM signalling pathway interacts tightly with cytoskeleton system. DNA repair protein ATM can regulate F‐actin protein remodelling in the cytoskeleton of A549 lung cancer cells.^9^, ^10^ ATM signalling pathway can adjust the cell cycle such as G1/S phase and G2/M phase cell cycle progression. Once the G2/M phase was blocked in the cells induced by internal or external factors, the actin and microtubule structure would break.^11^ Microtubule‐dependent sorting is essential for the subcellular localization of many organelles, vesicles, macromolecules and pathogens. Studies indicate that microtubules mediate intracellular sorting of NLRP3, and microtubules regulate assembly of the NLRP3 inflammasome.^12^ To be specific, assembly of NLRP3 inflammasome requires microtubules to induce the proximity of ASC and NLRP3. However, microfilaments (F‐actin) inhibit NLRP3 inflammasome activity and interact with NLRP3 and ASC.

Histone deacetylase inhibitors are a class of compounds that inhibit the activity of HDACs. They are emerging as promising chemotherapeutic agents which can inhibit tumour cell proliferation and regulate cell apoptosis.^13^ Zhou et al have revealed that HDAC inhibitor chidamide shows anti‐tumour effects by activating ATM signalling pathway.^14^ HDAC inhibitor Trichostatin A can regulate microtubule cytoskeleton remodelling and induce tubulin acetylation.^15^ Additionally, our previous studies have proved that HDACs inhibitors offer a potent hepatoprotective effect in ALF.^16^ In this study, D‐galactosamine (D‐Gal), lipopolysaccharide (LPS) and tumour necrosis factor‐α (TNF‐α) were applied to stimulate mice and the normal human liver cell line L02 cell to construct a mouse and cellular model of ALF. The HDAC inhibitor ACY1215 was administered to these ALF models to evaluate its protective effect. The ATM inhibitor KU55933 and F‐actin inhibitor cytochalasin B were also used in this study. The level of key molecules of ATM signalling, cytoskeletal protein F‐actin and vinculin and NLRP3 inflammasome were detected to explore the potential protective mechanisms of ACY1215 in ALF.

## 2 MATERIALS AND METHODS

### 2.1 Reagents and antibodies

ACY1215, KU55933 and cytochalasin B were purchased from Selleck. D‐Gal, LPS and TNF‐α were obtained from Sigma. Dulbecco's modified eagle medium (DMEM) basic and foetal bovine serum (FBS) were obtained from Gibco. Rabbit anti‐mice/human ATM, γ‐H2AX, p53, vinculin, NLRP3, IL‐1β, IL‐18 were purchased from Cell Signaling Technology. Rabbit anti‐mice/human Chk2, p21, ASC, GAPDH were purchased from Proteintech. Mice anti‐mice/human F‐actin were obtained from Abcam Co., UK. Caspase‐1 antibody was obtained from Santa Cruz Biotechnology. Secondary antibodies applied were the goat anti‐rabbit/mouse fluorescent antibody obtained from LI‐COR Biosciences, Inc.

### 2.2 Animal model preparation

Adult‐specific pathogen‐free (SPF) male C57BL/6 mice (n = 104) were purchased from Experimental Animal Center of Wuhan University. This study was approved by the Institutional Animal Care and Use Committee of Renmin Hospital of Wuhan University. All animals were adapted to the laboratory environment for 5 days before experimentation. All mice were kept at temperature (22 ± 2ºC) with 12‐h light/dark cycle and provide with food and water ad libitum. First, all mice were randomly divided into two large groups. The one group had 48 mice. The 42 mice were injected intraperitoneally with D‐Gal (400 mg/kg) and LPS (100 μg/kg). They were sacrificed, respectively, at 3 h, 6 h, 12 h, 24 h and 48 h after administration of D‐Gal/LPS. The six mice were given with same amount of normal saline. The other large group which had 56 mice were randomly divided into seven small group: normal, model, ACY1215, KU55933, ACY1215 + KU55933, cytochalasin B, ACY1215 + cytochalasin B group. Except for the normal group, the other six groups of mice were injected intraperitoneally with D‐Gal (400 mg/kg) and LPS (100 µg/kg) to induce the ALF model. ACY1215 (25 mg/kg), KU55933 (5 mg/kg) and cytochalasin B (10 mg/kg) were given, respectively, to the ACY1215, KU55933 and cytochalasin B group 2 h before ALF model implement. ACY1215 (25 mg/kg) and KU55933 (5 mg/kg) were used concurrently 2 h before ALF model implemented in ACY1215 + KU55933 group. ACY1215 (25 mg/kg) and cytochalasin B (10 mg/kg) were applied in ACY1215 + cytochalasin B group 2 h before ALF model implemented. After 24 h, the mice liver tissues were removed and serum was collected.

### 2.3 Histological studies and biochemical tests

The fresh liver specimens were fixed in 10% neutral‐buffered formalin for 1 day. Liver sections of each mice were stained with H&E to evaluate the histopathological changes. The serum alanine transaminase (ALT) and aspartate aminotransferase (AST) levels were detected by automated Aeroset chemistry analyzer.

### 2.4 TUNEL assay

Liver tissues from mice were deparaffinized with xylene and hydrated with 100%, 95%, 90%, 80% and 70% grade ethanol. 3% hydrogen peroxide in methanol was used to quench endogenous peroxide activity. Then, they were immersed in 0.1% Triton X‐100 solution for 3 min. 50 μl TUNEL reaction mix was dropped on the tissue for 50 min. Conversion agent‐POD of 50 μl was added and wiped dry. Incubation for 30 min at 37ºC was followed by DAB chromogen for 3 min. When wiped dry, the liver sections were counterstained with hematoxylin and mounted in neutral balsam. Finally, they were analysed under BX 51 light microscope (Olympus).

### 2.5 Cell culture and drug intervention

L02 cells were cultured in DMEM mixed with 10% FBS in the incubator within the 37ºC, 5% CO_2_ concentration and saturated humidity environment. The cell experiment was divided into seven groups: normal, model, ACY1215, KU55933, ACY1215 + KU55933, cytochalasin B, ACY1215 + cytochalasin B group. Except for the normal group, the other six groups were stimulated with TNF‐α (100 ng/ml) and D‐Gal (44 µg/ml) to induce the ALF model. ACY1215(2.5 μm), KU55933(10 μm) and cytochalasin B (5 μm) were added respectively in ACY1215, KU55933 and cytochalasin B group 2 h before given D‐Gal and TNF‐α. ACY1215 (2.5 μm) and KU55933 (10 μm) were used concurrently 2 h before model implemented in ACY1215 + KU55933 group. ACY1215 (2.5 μm) and cytochalasin B (5 μm) were administrated concurrently 2 h before giving D‐Gal/TNF‐α in ACY1215 + cytochalasin B group. After 24 h, all cells were harvested.

### 2.6 Immunofluorescence detection

Circular slides were placed in 24‐well plates and were seeded with 1 × 10^4^ L02 cells/500 µl. After treatments, the slides were fixed with 4% paraformaldehyde for 25 min. Then, the cells were permeabilized with 0.2% Triton X‐100 for 20 min, blocked with 5% BSA for 30 min. The cells were incubated with FITC and Cy3‐labelled fluorescent secondary antibodies for 1 h in dark environment. Finally, the cells were stained with DAPI for 5 min and observed under the fluorescence microscope. Mice liver tissues paraffin sections were dewaxed and hydrated, and immersed in citrate buffer for antigen retrieval. The next step is same with method of L02 cell immunofluorescence detection. Then, they were observed by a laser scanning confocal microscope (Olympus).

### 2.7 Western blotting

The liver tissues or L02 cells were homogenized by radio immunoprecipitation assay (RIPA) lysis buffer on ice to extract total protein. Protein concentration was detected by BCA protein assay reagent. Protein lysates (30 µg) were subjected to 10% SDS‐PAGE. After electrophoresis and electrical transfer, the proteins were transferred to PVDF membranes. After incubating with blocking buffer for 1 h, the membranes were incubated with primary antibodies against ATM (1:1000), γ‐H2AX (1:1000), Chk2 (1:1000), p53 (1:1000), p21 (1:1000), F‐actin (1:1000), vinculin (1:1000), NLRP3 (1:1000), ASC (1:1000), caspase‐1 (1:1000), IL‐1β (1:1000), IL‐18 (1:1000) and GAPDH (1:2000) overnight at 4˚C. The IRDye800CW secondary antibody (1:10,000) was incubated at 4˚C for 1 h. The protein bands were analysed using the Odyssey Infrared Imaging System (version 3.0, LI‐COR Biosciences).

### 2.8 Statistical analysis

Statistical analysis was performed using SPSS 16.0. Groups were compared by one‐way analysis of variance (ANOVA) or Student's *t* test. Data are reported as mean ± standard deviation. Statistical significance was considered at a *p* value < 0.05.

## 3 RESULTS

### 3.1 The histopathological changes of liver and level of ALT/AST in mice with D‐Gal/LPS‐induced ALF

**FIGURE 1:**
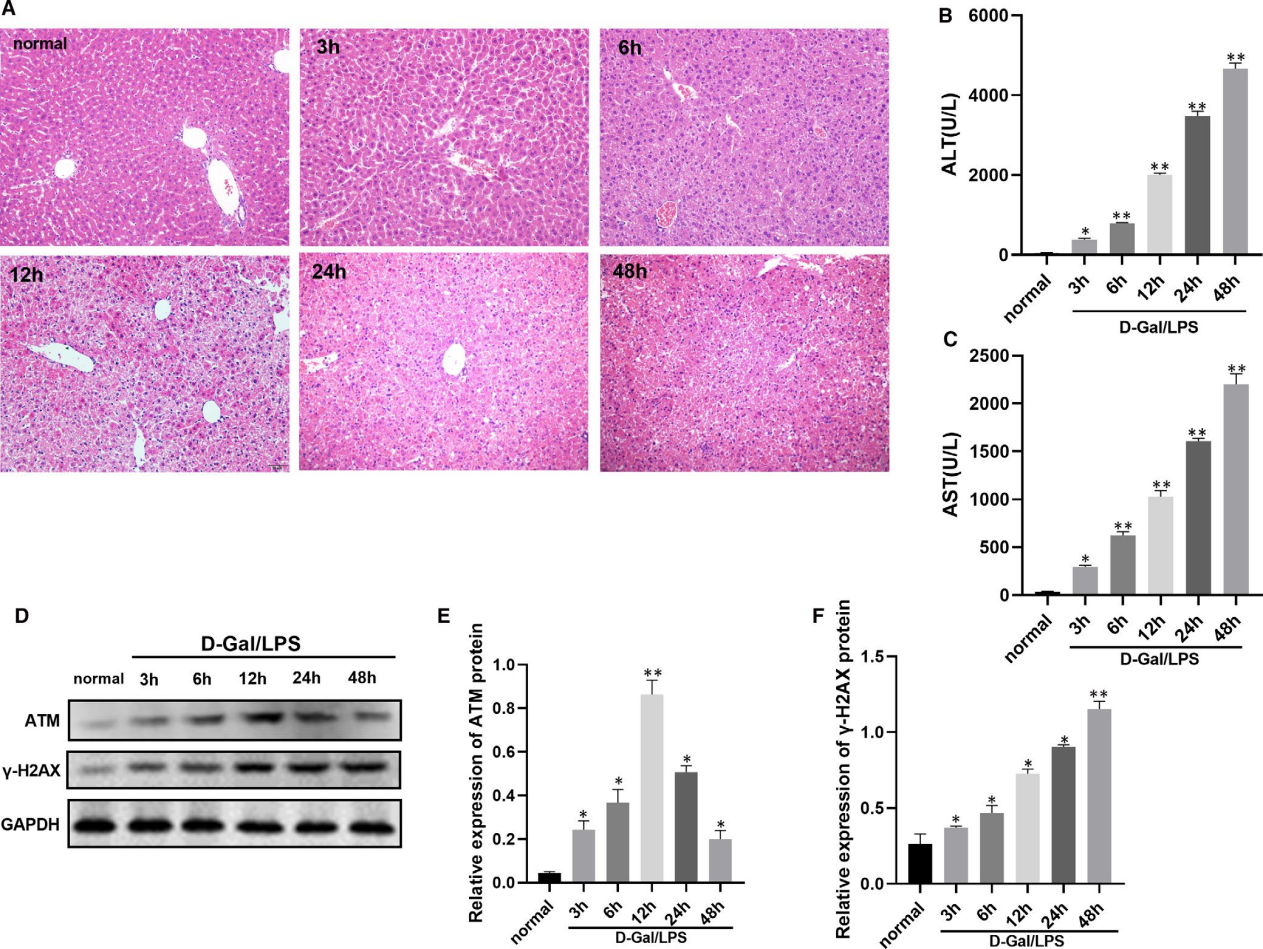
The pathological changes, liver function and protein level of ATM and γ‐H2AX in mice induced by D‐Gal/LPS at different time‐points. (A) Pathological changes in the mice liver tissue were detected by HE staining. (B‐C) The serum levels of ALT and AST in mice. (D‐F) Protein level ATM and γ‐H2AX in mice. Compared with normal group, *P < .05; Compared with normal group, **P < .01

As shown in Figure 1A, LPS/D‐Gal induced evident pathologic hepatic injury including disturbed architecture, hepatocyte necrosis, haemorrhage and neutrophil infiltration. As illustrated in Figure 1B‐C, compared with the normal group, treatment of D‐Gal/ LPS dramatically augmented the serum AST and ALT levels which these increases in a time‐dependent manner (*P* < .05).

### 3.2 The level of ATM and γ‐H2AX in mice with D‐Gal/LPS‐induced ALF

The protein level of ATM was significantly increased at all time‐points after D‐Gal/LPS stimulation with a maximal increase at 12 h (Figure 1D and 1E). Besides, the protein level of γ‐H2AX was remarkably elevated in liver tissue from mice injected with D‐Gal/LPS at the 3‐48‐h time‐points, which increased expression showed time dependence (Figure 1D and 1F).

### 3.3 ACY1215 ameliorated liver pathological damage, apoptosis rate and serum biochemical indicator in ALF mice

**FIGURE 2:**
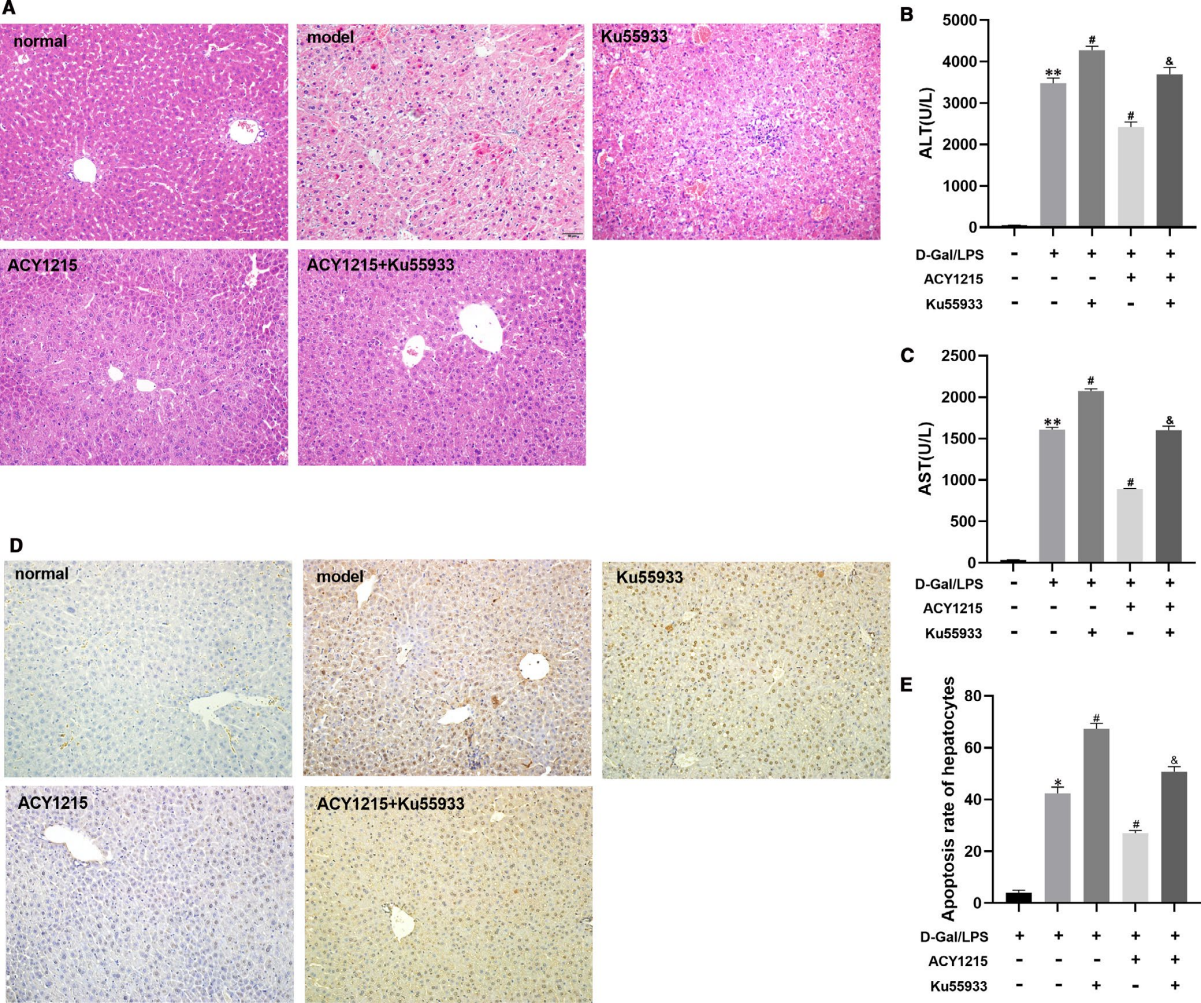
KU5933 reduced the protective effect of ACY1215 on ALF, manifested as aggravated liver pathological injury, increasing serum ALT, AST and hepatocellular apoptosis rate in ALF mice. (A) Pathological changes in the mice liver tissue at different group. (B‐C) The serum levels of ALT and AST in mice at different group. (D) Hepatocellular apoptosis rate in mice at different group. Compared with normal group, *P < .05; Compared with model group, ^#^ *P* < .05; Compared with ACY1215 group, ^&^ *P* < .05

As shown in Figure 2A, the hepatocytes were significantly necrotic and the hepatic lobular structure was blurred in model group. Moreover, the damage of liver structure was the most remarkable in KU55933 group. However, compared with model group, the structure of hepatic lobules in the ACY1215 group was clearer, and necrosis of hepatocytes was largely reduced. The ATM inhibitor KU55933 aggravated the liver pathological damage in ACY1215 group. As shown in Figure 2B and C, the serum ALT and AST in ALF mice were significantly elevated compared with normal group (*P* < .05). Compared with the model group, the ALT and AST levels were significant decreased in ACY1215 group (*P* < .05). KU55933 increased the serum ALT and AST levels in ACY1215 group (*P* < .05). As shown in Figure 2D and 2E, the apoptosis rate of hepatocytes in the KU55933 group was significantly increased, when compared with the model group (*P <* .05). ACY1215 decreased the apoptosis rate of hepatocytes in model group and in KU55933 group (*P* < .05).

### 3.4 ACY1215 activated ATM signalling pathway and increased the cytoskeletal protein level of F‐actin and vinculin in ALF mice and L02 cell

**FIGURE 3:**
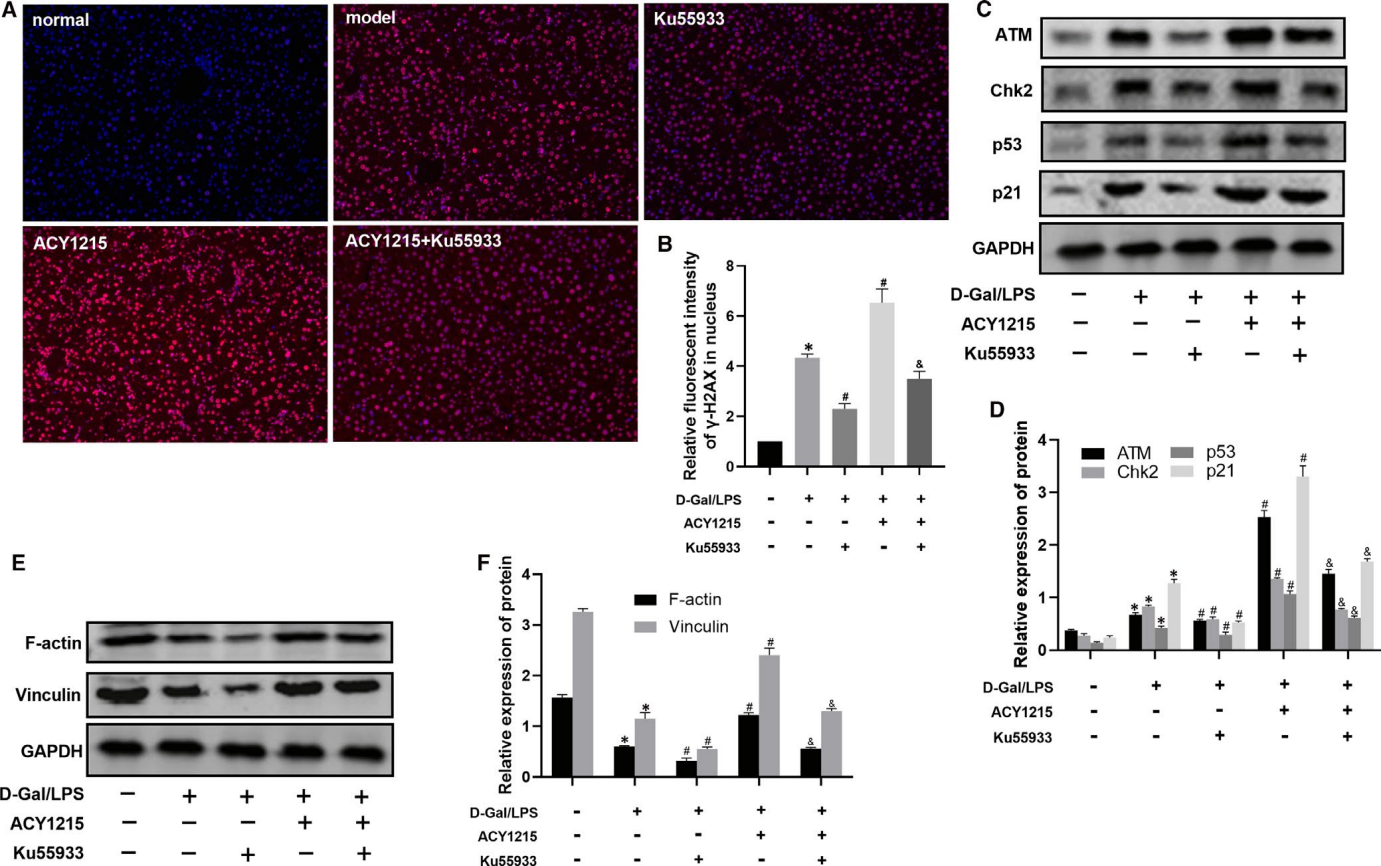
ACY1215 activate the ATM signalling pathway and increased protein levels of F‐actin and vinculin in ALF mice. (A‐B) The level of γ‐H2AX in liver tissue observed by fluorescence microscope. (C‐D) The protein levels of ATM, Chk2, p53 and p21 in liver tissue at different group. (E‐F) The protein levels of F‐actin and vinculin in liver tissue at different group. Compared with normal group, *P < .05; Compared with model group, ^#^ *P* < .05; Compared with ACY1215 group, ^&^ *P* < .05

**FIGURE 4:**
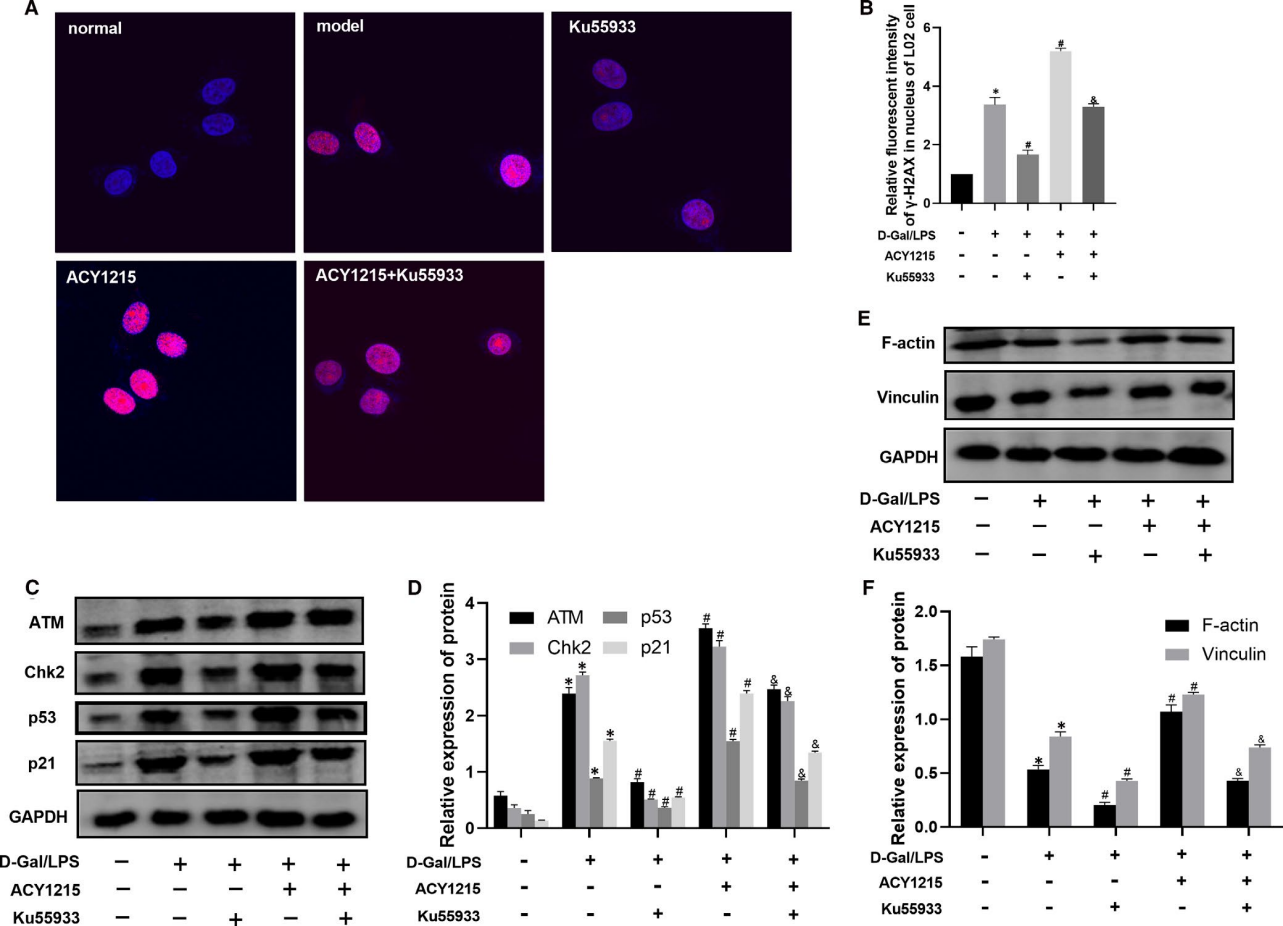
ACY1215 enhanced the ATM signalling pathway and elevated protein levels of F‐actin and vinculin in L02 cells stimulated by D‐Gal/TNF‐α. (A‐B) The level of γ‐H2AX in L02 cells observed by confocal microscopy. (C‐D) The protein levels of ATM, Chk2, p53 and p21 in L02 cells at different group. (E‐F) The protein levels of F‐actin and vinculin in L02 cells at different group. Compared with normal group, *P < .05; Compared with model group, ^#^ *P* < .05; Compared with ACY1215 group, ^&^ *P* < .05

As shown in Figure 3A‐B and Figure 4A‐B, compared with normal group, the level of γ‐H2AX was significantly increased in model group (*P* < .05). ACY1215 could further elevate the level of γ‐H2AX in model group (*P* < .05). However, KU55933 reduced the level of γ‐H2AX in ACY1215 group. As shown in Figure 3C‐D and Figure 4C‐D, compared with normal group, the protein level of ATM, Chk2, p53 and p21 was significantly increased in model group (*P* < .05). But ACY1215 pretreatment could further uplift protein level of ATM, Chk2, p53 and p21 in models. The ATM inhibitor KU55933 could decrease the protein level of ATM, Chk2, p53 and p21 in model and ACY1215 group (*P* < .05). As shown in Figure 3E‐F and Figure 4E‐F, compared with normal group, the protein level of F‐actin and vinculin was decreased in model group. ACY1215 increased the protein level of F‐actin and vinculin in model group (*P* < .05). However, the KU55933 lowered the protein level of F‐actin and vinculin in ACY1215 group (*P* < .05).

### 3.5 The F‐actin inhibitor cytochalasin B aggravate liver pathological damage and inhibit the cytoskeletal protein level of F‐actin and vinculin in ALF mice and L02 cell

**FIGURE 5:**
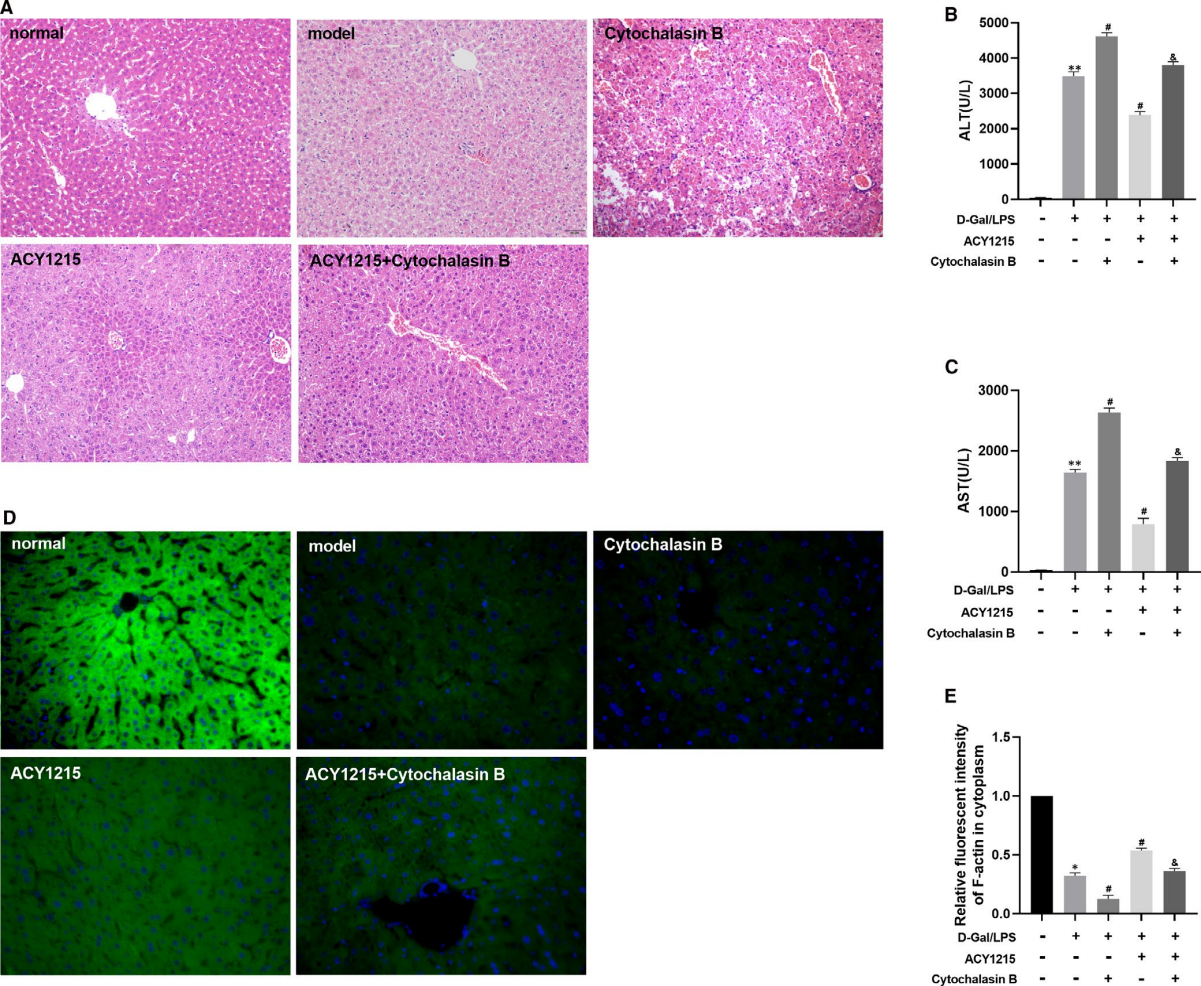
Cytochalasin B lessened the protective effect of ACY1215 on ALF, presented as aggravated liver pathological damage and increasing serum ALT and AST. (A) Pathological changes in the mice liver tissue at different group. (B‐C) The serum levels of ALT and AST in mice at different group. (D‐E) The level of F‐actin in liver tissue observed by fluorescence microscope. Compared with normal group, *P < .05; Compared with model group, ^#^ *P* < .05; Compared with ACY1215 group, ^&^ *P* < .05

**FIGURE 6:**
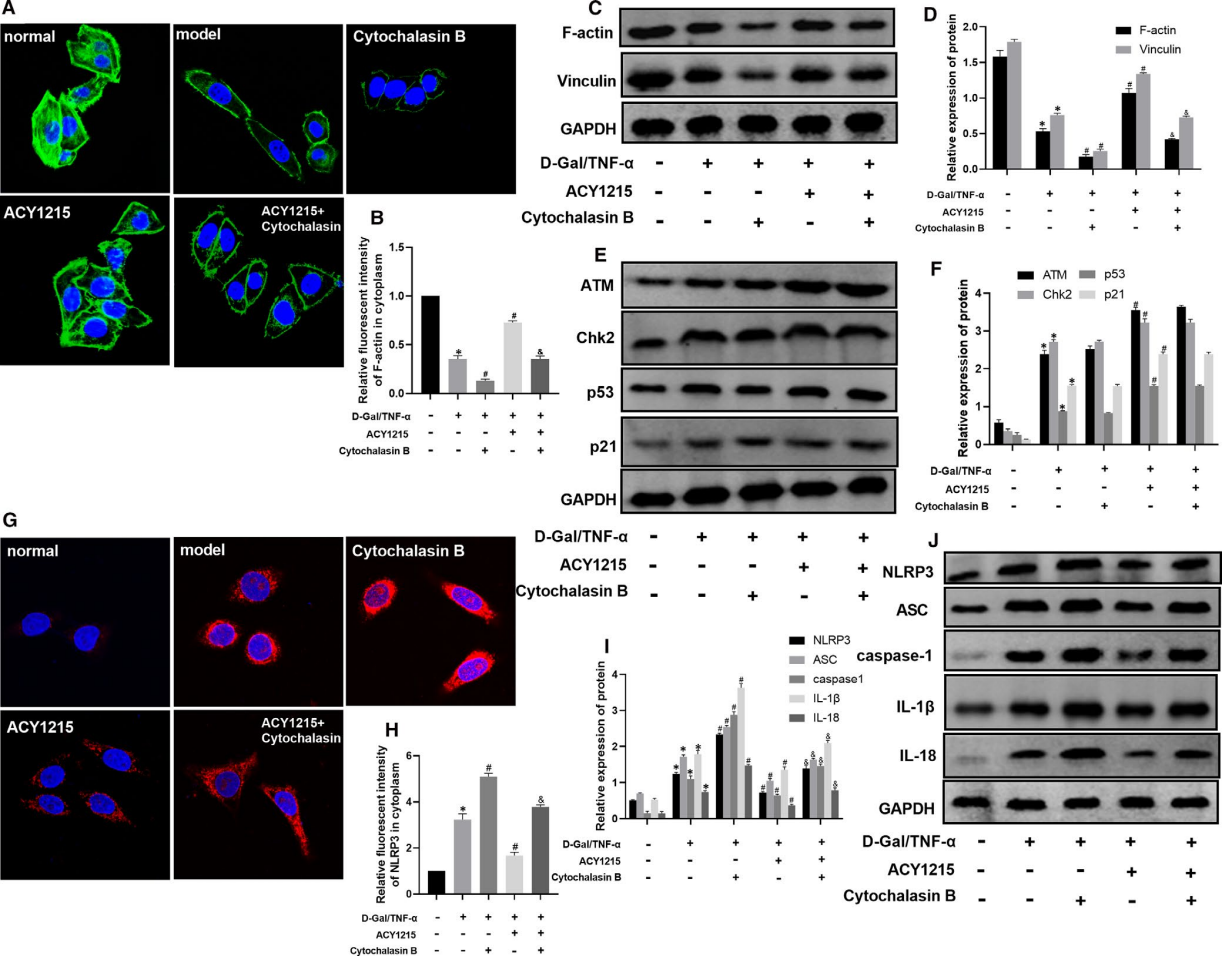
Cytochalasin B inhibits the protein level of F‐actin and vinculin and increased NLRP3 inflammasome level, but had no effect on ATM signalling pathway in ACY1215 group in L02 cell. (A‐B) The level of F‐actin in L02 cells observed by confocal microscopy. (C‐D) The protein levels of F‐actin and vinculin in L02 cells at different group. (E‐F) The protein levels of ATM, Chk2, p53 and p21 in L02 cells at different group. (G‐H) The level of NLRP3 in L02 cells observed by confocal microscopy. (I‐J) The protein level of NLRP3, ASC, caspase‐1, IL‐1β and IL‐18 in L02 cells at different group. Compared with normal group, *P < .05; Compared with model group, ^#^ *P* < .05; Compared with ACY1215 group, ^&^ *P* < .05

**FIGURE 7:**
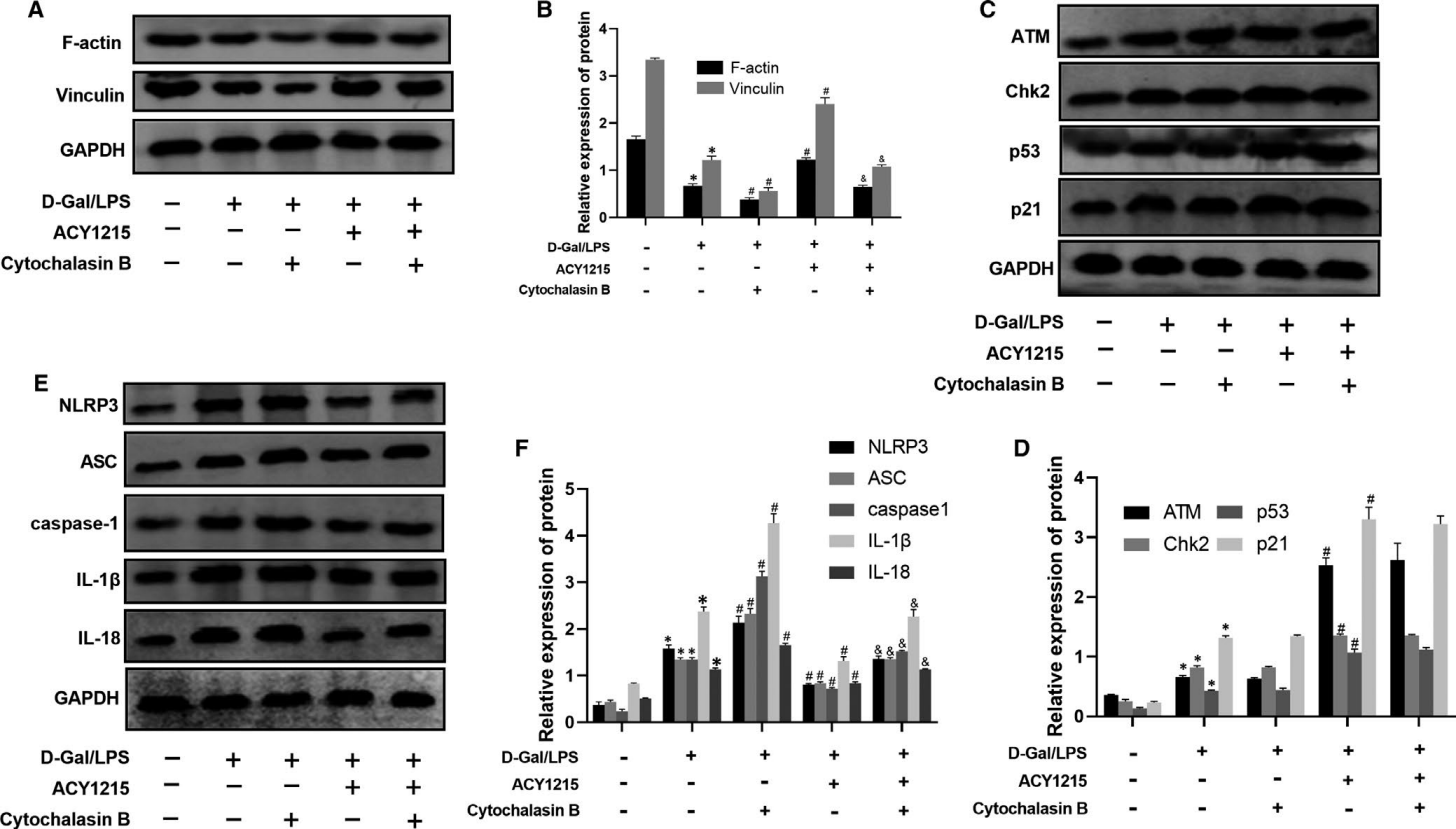
The protein level of ATM signalling pathway, cytoskeletal protein (F‐actin and vinculin) and NLRP3 inflammasome protein in model mice administrated with ACY1215 and or cytochalasin B. (A‐B) The protein level of F‐actin and vinculin in mice at different group. (C‐D) The protein level of ATM, Chk2, p53 and p21 in mice at different group. (E‐F) The protein level of NLRP3, ASC, caspase‐1, IL‐1β and IL‐18 in mice at different group. Compared with normal group, *P < .05; Compared with model group, ^#^ *P* < .05; Compared with ACY1215 group, ^&^ *P* < .05

As shown in Figure 5A, compared with model group, the normal structure of the liver was ruined and massive necrosis was more obvious in cytochalasin B group. Besides, the serum level of ALT and AST was significantly increased in cytochalasin B group compared with model group (*P <* .*05*). Cytochalasin B could also increase the serum level of ALT and AST in ACY1215 group (*P* < .05) (Figure 5B‐C). The immunofluorescence of liver tissue and L02 cell showed that level of F‐actin in cytochalasin B group was significantly reduced, when compared with model group, ACY1215 could increase the level of F‐actin in model and cytochalasin B group (*P* < .05) (Figure 5D‐E and Figure 6A‐B). There was a similar trend in the level of F‐actin and vinculin protein expression (*P* < .05) (Figure 6C‐D and Figure 7A‐B).

### 3.6 Cytochalasin B had no effect on ATM signalling pathway in ALF mice and L02 cell

As shown in Figure 6E‐F and Figure 7C‐D, the protein level of ATM, Chk2, p53 and p21 between model group and cytochalasin B had no statistical significance. Besides, cytochalasin B could not affect the protein level of ATM, Chk2, p53 and p21 in ACY1215 group (Figure 6E‐F and Figure 7C‐D).

### 3.7 ACY1215 inhibited the NLRP3 inflammasome in ALF mice and L02 cell

As shown in Figure 6I‐J and Figure 7E‐F, compared with normal group, the protein level of NLRP3, ASC, caspase‐1, IL‐1β and IL‐18 was remarkably increased in model group (*P* < .05). Compared with model group, the protein level of NLRP3, ASC, caspase‐1, IL‐1β and IL‐18 was significantly reduced in ACY1215 group (*P* < .05). However, those proteins level were higher in ACY1215 + cytochalasin B group when compared with ACY1215 group (*P* < .05). Besides, the immunofluorescence of L02 cell showed that the level of NLRP3 presents a similar trend with the protein level of NLRP3, ASC, caspase‐1, IL‐1β and IL‐18 (*P* < .05) (Figure 6G‐H).

## 4 DISCUSSION

Acute liver failure is a life‐threatening and complicated disease that has a high mortality.^17^ Furthermore, there is lack of efficient drugs for treating ALF.^2^ Studies have found that in the animal model of ALF, the complete double‐stranded DNA structure in the injured hepatocyte is broken.^18^ In addition, the damage of double‐stranded DNA of hepatocyte will continuously deteriorate in ALF. γ‐H2AX is formed by phosphorylation of the 139th serine on the H2AX histone after double‐stranded DNA scission occurs in the cell.^19^ It is known as an early marker of double‐stranded DNA breaks. After double‐stranded DNA breaks caused by exogenous or endogenous factors, γ‐H2AX has a potent repair effect. It can block the cell cycle, promote DNA repair and maintain gene stability.^20^ Our data indicated that D‐Gal combine with LPS lead to the severe liver injury which characterized with remarkable liver histopathological damage and increased serum level of ALT and AST. Besides, the level of γ‐H2AX in model mice and L02 cells is significantly elevated. And in animal model, the expression of γ‐H2AX is constantly increased at the 3‐48 h after administration of D‐Gal and LPS. The results indicated that D‐Gal/LPS could induce the destruction of complete double‐stranded DNA structure in hepatocytes and improve the level of γ‐H2AX to relieve this damage in ALF.

The DNA repair and cell cycle checkpoint pathways belong to DNA damage response (DDR), which allow cells to address various sources of DNA damage.^21^ The ATM gene was identified by positional cloning and encodes a 3056 amino acid protein.^22^ Laura et al have demonstrated that ATM deficiency exacerbates cardiac remodelling late post‐myocardial infarction with effects on cardiac function, fibrosis, apoptosis and myocyte hypertrophy.^23^ Consistent with the critical roles of ATM protein in DNA repair and cell cycle, it has multifarious substrates such as Chk2 and p53.^24^, ^25^ Our data showed that in ALF model mice and L02 cell, the level of ATM, Chk2, p53 and p21 was increased. Notably, the ATM inhibitor KU55933 could exacerbate liver damage and decrease the level of ATM, Chk2, p53 and p21 in ALF mice and L02 cell. Those results indicated that those increased proteins could promote themselves against DNA damage caused by D‐Gal and LPS or TNF‐α. Once the ATM signalling pathway was blocked, the liver damage would be aggravated.

In the process of ALF, there is a large number of necrosis and apoptosis of hepatocytes.^26^ Moreover, the cytoskeleton of hepatocytes is damaged as well. The cytoskeleton is protein fibre network structure in the cells which is composed of microtubules, intermediate fibres and microfilaments.^27^ Actin is a vital skeletal protein that belongs to cytoskeleton structure. On the one hand, it maintains the normal shape of the cell. On the other hand, it can regulate cell stress fibre formation, adhesion, migration, apoptosis and transmembrane information transmission. Actin exists in two forms: soluble monomeric globular actin (G‐actin) and polymeric fibrous actin (F‐actin). Only F‐actin has biological activity, and the two forms of actin can be transformed into each other.^28^ Vinculin is a cytoskeletal protein involved in the formation of adhesion junctions.^29^ Its function is to anchor F‐actin. Our results also indicate that the level of cytoskeleton protein F‐actin and vinculin was remarkably decreased in ALF.

Histone deacetylase inhibitors are being developed as promising cancer therapeutics.^13^ They possess various effects such as anti‐inflammatory and antioxidant. At present, they are studied as many non‐neoplastic diseases including non‐alcoholic fatty liver disease, pulmonary fibrosis and heart failure.^30^ And in present study, histone deacetylase inhibitor ACY1215 was selected to be intervention agent to observe itself potential protective effect in ALF.

Our data showed that ACY1215 could reduce liver pathological damage, apoptosis rate and the serum level of ALT and AST in ALF mice. ACY1215 increased the level of ATM, Chk2, p53, p21 and γ‐H2AX in ALF. But the ATM inhibitor KU55933 aggravated liver damage in ALF. Furthermore, The KU55933 lessened the protective effect of ACY1215 on ALF via decreasing the level of ATM, Chk2, p53, p21 and γ‐H2AX. The previous study indicated that the ATM signalling pathway has a tight connection with cell cytoskeleton system. Of note, our study found that KU55933 could the downregulate level of F‐actin and vinculin in ALF. But the F‐actin inhibitor cytochalasin had no effect on the level of ATM, Chk2, p53 and p21 in ALF. The results indicated that ATM signalling pathway may act as the upstream signalling pathway to regulate the cytoskeleton proteins F‐actin and vinculin in ALF.

Abundant previous reports have showed that the inhibition of NLRP3 inflammasome may contribute to ameliorate of ALF.^31^ And in our study, we found that the level of NLRP3 inflammasome was increased in ALF which was consistent with previous results. We found that ACY1215 could inhibit the level of NLRP3 inflammasome to protect against liver injury in ALF. Besides, Qingsong et al indicated that HDAC6 inhibitor Cay10603 can improve the high glucose‐induced oxidative stress and inflammation in retinal pigment epithelial cells via regulating the NLRP3 inflammasome.^32^

Moreover, previous researchers have revealed that cytoskeleton F‐actin can regulate NLRP3 inflammasome activity.^33^ To be specific, suppressing F‐actin can accentuate the activity of NLRP3 inflammasome to increase IL‐1β production. Our results showed that the F‐actin inhibitor cytochalasin B not only further exacerbate liver injury but increase the level of NLRP3 inflammasome in ALF. And cytochalasin weakened the protective effect of ACY1215 on ALF via decreasing the level of F‐actin and vinculin and increasing the expression of NLRP3, ASC, caspase‐1, IL‐1β, and IL‐18.

In summary, the role of NLRP3 inflammasome has been studied in many types of liver diseases and has become an important target for drug design and development. Based on our data, we proved that the NLRP3 inflammasome in ALF is mitigated by HDAC6 inhibitor ACY1215. Additionally, our results confirm the therapeutic molecular role of ACY1215 by regulating ATM/F‐actin signalling pathway in ALF. However, further investigation is needed to determine whether ACY1215 can be clinically applicable for treating hepatic diseases.

## CONFLICT OF INTEREST

The authors declare that they have no conflicts of interest.

## AUTHOR CONTRIBUTION

**Qian Chen:** Formal analysis (equal); Methodology (equal); Writing‐original draft (equal). **Yao Wang:** Formal analysis (equal); Methodology (equal); Resources (equal). **Fangzhou Jiao:** Methodology (equal); Visualization (equal). **Pan Cao:** Methodology (equal); Visualization (equal). **Chunxia Shi:** Formal analysis (equal); Methodology (equal); Resources (equal). **Maohua Pei:** Formal analysis (equal); Methodology (equal). **Luwen Wang:** Resources (equal); Validation (equal). **Zuojiong Gong:** Conceptualization (lead); Project administration (lead); Supervision (lead); Writing‐original draft (lead); Writing‐review & editing (lead).

## Data Availability

The data that support the findings of this study are openly available in [repository name, eg ‘figshare’] at reference number.
